# Habits

**Published:** 1902-04-26

**Authors:** 


### The Hospital

A Journal of

The Medical Sciences and Hospital Administration.

Vol. XXXII.—No. 813.] Edited by Sir Henry Burdett, K.C.B., and Solomon C. Smith, M.D., M.R.C.P. [April 26, 1902.

### "HABITS."

A curious expression of one of the tendencies of modern civilisation is furnished by the fact that, in the new edition of *Quain's Dictionary of Medicine* there appears, for the first time, an article on "Habit," as a general term applied to the practice of taking narcotics as pursued by people who, if not actually in health, have as yet no perceptible disease other than what would be described as a "craving" for this or that kind of stimulant or sedative. The introduction of the hypodermic syringe, the enlarged resources of modern pharmacy, and the publicity given to the prevalence of the "habit" in question by various writings, some avowedly of fiction, and others professing to be more or less records of fact, have, no doubt, been to a very large extent responsible for its increase; but its foundation seems to repose upon a demand which is almost universal among mankind. In every climate, and among every people, experience has taught the lesson that there are drugs which, in periods of storm and stress, are more prompt in their action and more restorative in their effects than food, and which, for a time at least, may even serve as substitutes for repose. It may almost be maintained, moreover, that the agents of this kind which have originally been employed by different peoples, have, on the whole, had some special adaptation to the needs of climate or of race; and so have been possibly more useful and less hurtful in the places of their origin than they have become when introduced into other countries. Opium, for example, is clearly better adapted than alcohol to the requirements of Chinese, Hindoos, or Malays, and the precise opposite would probably be true of the majority of the inhabitants of cold or temperate climates. But between the occasional employment of any such agents in special circumstances of requirement, and their habitual use as mere luxuries, it is probable that a very broad distinction should be drawn; and few subjects of inquiry could be of greater interest to the physiologist, or to the pathologist whose inquiries have chiefly been addressed to altered conditions of the nervous system, than to ascertain the manner and extent in which the conditions and capabilities of that system are liable to be permanently modified by the constant use of drugs which, manifestly and to the perceptions of all men, do daily modify its modes of operation for longer or shorter periods. Everybody is familiar with the fact that the effects of alcohol, for example, which at first and for a considerable time may be regarded as merely nutritional, and as limited to the production of some change in the conditions under which customary functions are performed, with a corresponding but merely temporary change in the intensity or in the precise character of their performance, do after a time become structural, and such that the modification becomes permanent. These structural changes are not limited, in the case of alcohol, to the brain, but they affect all the organism. "Alcohol," wrote Dr. Moxon, in describing the sot, "has left unchanged nothing of him worth speaking of," and it is just this fact that creates the supreme difficulty of dealing with him, and renders worthless his "intervals of sottish repentance." May we not conclude that the modern prevalence of "habits" is mainly due to the fact that we have largely abused the good gifts of nature by employing, as daily luxuries, and in time as artificially-created necessaries, things which, if kept in their right places, would have been reserved as resources in emergency or in distress. We are told by those who take it that tobacco is an eminently tranquillising and soothing agent, capable of softening down many of the asperities of life; but it is manifest that it can no longer exert this influence, or fulfil this purpose, in the case of a consumer who is never long without it, even when the ways which he is called upon to traverse are entirely smooth. If difficulties come to him the tobacco fails of its effect, and something more powerful, or to which he is less habituated, must be—or at least too often is—resorted to. We are strongly inclined to the belief that the excessive smoking of our time, under conditions which do not seem to afford any sufficient justification for it, must to some extent be blamed for the manifestly increasing prevalence of "habits," and that, if the comparatively mild and harmless drug were used discreetly and in moderation, the necessity or fancied necessity for the stronger and more harmful one would be far less likely to assert itself. As a matter of fact, the absolute need for either one or the other can only seldom arise in the conditions of modern life, and the daily use of the narcotic can hardly be looked upon in any other light than as a piece of unnecessary self-indulgence. That this is so is abundantly shown by the example of women, who smoke but rarely, and whose want of a panacea against the smaller ills of life, the worries of a household, the cares incidental to the possession of children and servants, is certainly far greater than that of their husbands. Possibly many women who have now fallen into "habits" might have been preserved from them if they had smoked; and it seems certain that many men would have been preserved from them if they had used tobacco without abusing it.

